# Respiratory mechanics and ventilatory control in overlap syndrome and obesity hypoventilation

**DOI:** 10.1186/1465-9921-14-132

**Published:** 2013-11-20

**Authors:** Johan Verbraecken, Walter T McNicholas

**Affiliations:** 1 Department of Pulmonary Medicine and Multidisciplinary Sleep Disorders Centre, Antwerp University Hospital and University of Antwerp, Wilrijkstraat 10, Edegem 2650, Belgium; 2 Pulmonary and Sleep Disorders Unit, St. Vincent’s University Hospital, Dublin, Ireland; 3 Conway Institute of Biomolecular and Biomedical Research, University College Dublin, Dublin, Ireland

**Keywords:** Respiratory mechanics, Ventilatory control, COPD, Apnea, OHS, Respiratory drive, Neurohormonal

## Abstract

The overlap syndrome of obstructive sleep apnoea (OSA) and chronic obstructive pulmonary disease (COPD), in addition to obesity hypoventilation syndrome, represents growing health concerns, owing to the worldwide COPD and obesity epidemics and related co-morbidities. These disorders constitute the end points of a spectrum with distinct yet interrelated mechanisms that lead to a considerable health burden. The coexistence OSA and COPD seems to occur by chance, but the combination can contribute to worsened symptoms and oxygen desaturation at night, leading to disrupted sleep architecture and decreased sleep quality. Alveolar hypoventilation, ventilation-perfusion mismatch and intermittent hypercapnic events resulting from apneas and hypopneas contribute to the final clinical picture, which is quite different from the “usual” COPD. Obesity hypoventilation has emerged as a relatively common cause of chronic hypercapnic respiratory failure. Its pathophysiology results from complex interactions, among which are respiratory mechanics, ventilatory control, sleep-disordered breathing and neurohormonal disturbances, such as leptin resistance, each of which contributes to varying degrees in individual patients to the development of obesity hypoventilation. This respiratory embarrassment takes place when compensatory mechanisms like increased drive cannot be maintained or become overwhelmed.

Although a unifying concept for the pathogenesis of both disorders is lacking, it seems that these patients are in a vicious cycle. This review outlines the major pathophysiological mechanisms believed to contribute to the development of these specific clinical entities. Knowledge of shared mechanisms in the overlap syndrome and obesity hypoventilation may help to identify these patients and guide therapy.

## Introduction

**Figure 1:**
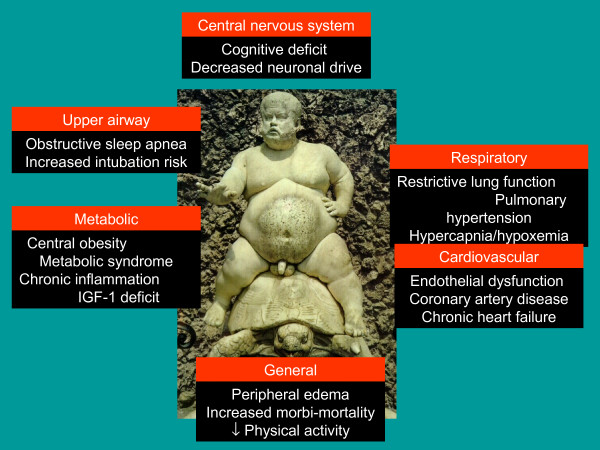
Central and peripheral consequences of obesity (statue of Bachus in Boboli Gardens, Firenze, Italy).

Over recent decades, our understanding of the mechanisms leading to sleep disordered breathing has steadily improved, with most studies focussing on ventilatory control mechanisms and upper airway patency during sleep. Instability of the breathing pattern can go along with an increase in upper airway resistance, increased collapsibility of the upper airway and poor coordination of local reflex mechanisms, which can result in obstructive apneas [1]. Interaction between obstructive sleep apnea (OSA), and a number of different distinct clinical categories, like COPD, chronic heart failure, neuromuscular disorders and obesity, can lead to more complex disorders, with complications sharing common pathways [1-3]. In obese subjects, respiratory system mechanics can become disturbed, in isolation or in association with upper airway pathology, and obesity hypoventilation syndrome (OHS) may develop as a result [4-6]. According to the International Classification of Functioning, OHS is a chronic condition associated with respiratory, metabolic, hormonal and cardiovascular impairments, leading to a decrease in daily life activities, a lack of social participation and a high risk of hospitalisation and death (Figure 1) [7]. OSA and COPD are both prevalent disorders [8-10], which are gaining more importance, due to the obesity epidemic in Western countries [11,12] in addition to smoking behaviour over previous decades [13]. The World Health Organisation (WHO) predicts that around 10% of the global population will be obese by 2015 [11,12]. Patients who share both disorders have inspiratory flow limitation on the one hand (OSA), and expiratory flow limitation on the other hand (COPD) [3,14]. This coincidence of complicated breathing will compromise sleep more than in stand alone disorders, with an accumulation of health risks and complications [15-17].

In this review we would like to address the pathophysiological interactions between COPD, sleep and OSA in the overlap syndrome as well as the mechanisms involved in the pathogenesis of OHS. Both OSA and OHS, besides central sleep apnea, take part of the spectrum of sleep-disordered breathing [18,19], with preserved or enhanced chemical drive on the one hand, and blunted chemical drives on the other hand. The majority of these patients presents with obesity. However, they are also quite distinct from each other in a sense that obesity is not always present in overlap syndrome, and chemical drive can be preserved, while OSA is not always present in obesity hypoventilation, and COPD is an exclusion criterion in the definition of OHS. Therefore, it was preferred to discuss them separately.

## Overlap syndrome

### Extent of the problem

Obstructive sleep apnea syndrome and chronic obstructive pulmonary disease (COPD) are two diseases that often coexist within an individual. This coexistence is referred to as the overlap syndrome and is the result of chance rather than a pathophysiological link. Previous studies have suggested that the prevalence of OSA in patients with COPD, and COPD in patients with OSA, was high, sometimes unexpectedly high [20-23]. It has been believed that the presence of COPD could predispose to the development of OSA, since the two conditions share some etiologic factors such as tobacco smoking. This remained an unresolved question up to very recent years. In 2003 the Sleep Heart Health Study provided solid epidemiologic data about the coexistence of COPD and OSA [24]. Participants with obstructive airway disease had significantly lower mean and median RDI than those without obstructive airway disease. However, after stratification by BMI quartile, RDI values were similar in the participants with and without obstructive airway disease.

We presently know that the prevalence of COPD is over 10% in adults 40 years of age or older, and may exceed 20% [9,10,25]. Accordingly, it does not appear that the prevalence of COPD is increased in patients with OSA, when compared with the general population.

If COPD is present in about 10% of the adult population 40 years of age or older, and if the prevalence of OSA in the same population is in the range of 5 to 10%, overlap syndrome can be expected to be present in 0.5 to 1% of the general population over 40 years of age, which represents a relatively prevalence [3]. The prevalence of COPD Gold stage III and IV, wherein chronic or intermittent hypoxemia and hypercapnia are more common, is in the range of 3% to 4% and likely represents the segment of the COPD population that can develop severe cardiopulmonary complications if coincidence with OSA [14,26].

### Pathophysiology of overlap syndrome

Overlap patients present sleep-disordered breathing associated to upper and lower airway obstruction and a reduction in respiratory drive. These patients present unique characteristics, which set them apart from either COPD or OSA patients. The overlap population tends to be older than the simple OSA population, with more frequent hypoxemia and hypercapnia, higher mean pulmonary artery pressures, but similar BMI [2]. Resta et al. showed that overlap patients had higher PaCO_2_, as well as similar AHI compared to OSA patients without COPD [27]. O’Brien and Whitman found that overlap patients were older and less obese than isolated OSA controls [28].

#### Association with body mass index and smoking

Several confounding factors may influence relationships between OSA and COPD, particularly smoking and BMI. Obesity is a cardinal feature in OSA, while low BMI is common in COPD, especially in patients with advanced disease [24,29]. This feature may protect against OSA, and is supported by the finding of a lower AHI in subjects with airflow obstruction, relating to lower BMI [30]. However, many other patients with COPD have elevated BMI, thus predisposing to OSA, and the finding of a higher AHI in overweight patients with airflow obstruction supports this possibility [24]. Smoking is a risk factor for OSA and COPD, and several reports found a higher AHI in smokers than nonsmokers [31]. In the Wisconsin Sleep Cohort Study it was found that an AHI of at least 5/hour was three times more likely in current smokers than in never-smokers. Heavy smokers (≥40 cigarettes/d) had an OR of 6.74 for AHI of at least 5/hour [32]. Cigarette smoking predisposes to OSA by increasing upper airway resistance due to local inflammation and edema [33]. Altogether, it seems that in the majority of the patients both obesity and smoking play an important role in the genesis of overlap syndrome.

#### Alterations in respiratory mechanics and ventilatory control during sleep related to COPD

**Figure 2:**
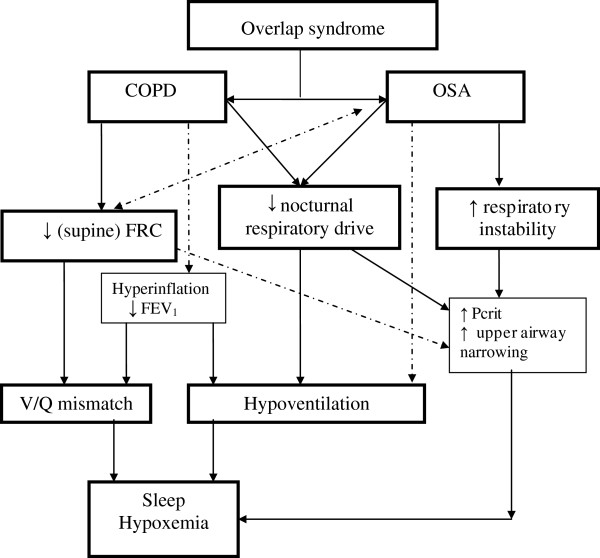
Mechanisms through which COPD and OSA interact during sleep.

Similar changes in respiratory function occur during sleep in patients with COPD as in healthy subjects, independent of OSA, although some can be more profound in COPD and are related to accentuated physiological adaptations, like hypoventilation [34,35] (Figure 2). It was shown that in patients with COPD, respiratory control centre output was reduced and upper airway resistance was increased, both most pronounced during REM sleep [36]. The circadian variation in airflow is exaggerated in patients with COPD [37-39], with a mean daily change in FEV_1_ and FVC of 30%. Moreover, the physiologic hypoventilation normally present during sleep is accentuated, resulting in greater falls in SaO_2_ during sleep, with associated hypercapnia. This is likely a result of the increased physiologic dead space in COPD that leads to an even greater decrease in alveolar ventilation with lower tidal volumes than in normal subjects. Because many COPD patients have awake hypoxemia, they are especially prone to nocturnal oxygen desaturation by being on the steep portion of the oxyhemoglobin dissociation curve [30,34]. In addition, there is reduced contractility of skeletal muscles, which includes the accessory muscles of respiration for adequate ventilation, and REM related muscle paralysis may result in poor ventilation during sleep. The function of the diaphragm is even more decreased than in healthy subjects due to stretching related to lung inflation, and therefore relatively inefficient [40], thus necessitating an increased accessory muscle contribution to breathing [41]. The relatively large dependency on diaphragm function during sleep in COPD, due to the accessory muscle weakness, and the dominant diaphragmatic activity during REM sleep, might explain why patients with loss of respiratory muscle strength during REM sleep show nocturnal oxygen desaturations [42]. In advanced COPD, skeletal muscle atrophy and dysfunction is common, which may further compromise the contribution made by accessory muscles [29]. In addition, ventilation-perfusion mismatch and a reduced FRC may also play a role in nocturnal hypoxemia in COPD [43]. Taken together, accentuated physiologic hypoventilation, increased physiologic dead space, lower awake oxygen levels, reduced contractility of respiratory muscles, lung inflation, (respiratory) muscle atrophy and ventilation-perfusion mismatch contribute to a certain extent to alterations in the ‘COPD’ component of the overlap syndrome.

#### Pathophysiological interactions between OSA and COPD

Several pathophysiological factors have been identified which influence the relationship between OSA and COPD. COPD-related factors that may predispose to OSA include rostral shift of peripheral edema when supine, resulting in fluid accumulation in the neck, thus contributing to pharyngeal narrowing [44]. Although no evidence is available in COPD, it might be particulary expected in patients with cor pulmonale, among whom peripheral edema is a major feature [45]. Peripheral edema can be present in the absence of right heart failure in COPD and is not diagnostic of cor pulmonale [46]. The pathogenesis of edema formation in COPD is complex. Renal blood flow is reduced, the renin-angiotensin system is activated, leading to increase in proximal renal tubular sodium reabsorption. Sodium retention is enhanced by hypercapnia and ameliorated by long-term oxygen therapy in hypoxemic patients [47]. As discussed earlier, BMI and smoking also affect pathophysiological relationships. Neck obesity contributes to upper airway narrowing, which is the key factor in OSA pathophysiology, and such narrowing has been related to nocturnal oxygen desaturation in COPD, independent of OSA [48]. A role could also be attributed to central obesity, which predisposes to OSA [49]. Its mechanical impact will be discussed in the obesity hypoventilation section. Finally, use of corticosteroids may influence interactions between COPD and OSA by promoting central obesity and fluid retention with associated upper airway narrowing, in addition to myopathy and metabolic alkalosis [30]. Among all these factors, it could be assumed that central obesity is the strongest predictor.

#### Ventilatory control in overlap

Very few studies have evaluated ventilatory control in overlap syndrome [50,51]. Radwan et al. [50] reported a higher breathing frequency and a lower tidal volume in overlap compared to OSA patients. OSA patients presented similar values to controls in awake ventilatory response to CO_2_ and occlusion pressure responses, while overlap patients had both blunted ventilatory responses and mouth occlusion pressure responses to CO_2_. The ventilatory responses could be disturbed by lung mechanics and gas exchange. In subjects with chronic hypercapnia, there is an increased blood bicarbonate concentration, which may inhibit the ventilatory response to CO_2_ and decreases mouth occlusion pressure response during wakefulness and sleep [50].

When normocapnic, overlap patients can however have a normal or even enhanced ventilatory response to CO_2_[51]. This is in contrast to the data on decreased hypercapnic (HCVR) and hypoxic (HVR) ventilatory response in OHS, as compared to obese, non-hypercapnic subjects [52]. Thus, obesity seems to be associated with compensatory mechanisms. If they fail, OHS may appear. Others have hypothesized that hyperinflation of the lung may also decrease mouth occlusion response [53]. During sleep factors related to decreased mouth occlusion pressure are respiratory muscle fatigue, related to the mechanical disadvantage of chest wall hyperinflation, and reduced FRC, related to supine posture and sleep state, as discussed earlier (Figure 2). Hypoxemia and hypercapnia are however less severe in patients with overlap than in patients with OHS [54]. Altogether, the wide range in chemical drives illustrates the existence of different phenotypes in overlap patients, but also reflects a gradual adaptation of chemoreceptors secondary to mild elevation of serum HCO3- that can occur even during acute hypercapnia.

### Pathophysiological consequences

Data about the complications of overlap syndrome are scarce, particularly for those patients who have less severe COPD. Currently, it is unclear if patients who have mild COPD are at risk for the same early complications as those who have more severe disease. Similarly, the significance of mild versus severe OSA is unknown as it relates to complications, course, and prognosis of the disease. Research in this area suffers from the so-called “iceberg phenomenon”, a metaphor emphasising that for virtually every health problem the number of known cases of disease is outweighed by those that remain undiscovered. Close to this is the “clinician fallacy”, in which an inaccurate view of the nature and causes of a disease results from studying the minority of cases of the disease that are seen in clinical treatment settings [55].

#### Disturbed sleep quality

Subjects with the overlap syndrome have higher Epworth sleepiness scores, lower total sleep time, lower sleep efficiency, and a higher arousal index compared with those with COPD alone [24]. However, there was only a small difference between those with OSA alone and those with the overlap syndrome. It seems that in patients with mild COPD, there is little effect on sleep quality. As COPD becomes more severe, however, there are increased sleep complaints. In addition, when OSA is superimposed on COPD, there are more sleep complaints and possibly more deleterious physiologic effects [37]. Recently, Kwon et al. reported that increased severity of hyperinflation, which is the ratio of inspiratory capacity to total lung capacity (IC/TLC), is associated with worse sleep efficiency in overlap, independent of apnea and nocturnal hypoxemia [56,57]. The mechanisms underlying this observation are uncertain, and the relationship was also preserved after controlling for bronchodilator medications. A potential mechanism for this association includes increased work of breathing due to lung hyperinflation, which, in turn, leads to greater difficulty with sleep onset and more sleep disturbances. Close to this observation, significantly elevated St. George Respiratory Questionnaire scores for total score and for each of the three components have been reported in overlap patients, as compared with patients who had isolated COPD [58]. Altogether, sleep disturbance is related to the relative contribution of each component of the overlap syndrome, whereas sleep fragmentation is most related to the severity of OSA.

#### More profound nocturnal oxygen desaturations and CO_2_ desensitisation

The worsening hypoxemia seen at night in COPD patients who do not have OSA is attributable to a combination of alveolar hypoventilation and ventilation-perfusion (V/Q) mismatch. Typically, these patients present with a pattern of chronic hypoxemia. Alveolar hypoventilation represents the predominant mechanism, especially when individuals are in REM sleep in which hypoventilation is common in normal controls [14]. Hypoxemia is also well described in OSA (Figure 2), but in this category, patients present with a pattern of intermittent hypoxemia. The hypoxemic events in these subjects are closely associated with apneas and hypopneas, and result from alveolar hypoventilation. Individuals with OSA have a normal oxygen saturation between respiratory events, unless significant V/Q mismatch or shunting takes place. Although a pathologic link between the two diseases may not exist, it is clear that when they coexist, patients may display more severe nocturnal oxygen desaturations and worsened sleep quality [21,24]. Oxygen saturation between apneas may typically remain low in patients with the overlap syndrome (and could be applied to OHS as well), although occasionally it may occur as well in other patients with severe OSA. Mechanisms through which hypoxemia and hypercapnia persist between apneas are complex, and include at least insufficient interapneic ventilation and temporal V/Q mismatch [59]. Lower FRC, and hence lower oxygen reserve, could explain, at least partly, the lower oxygen levels in the different conditions [60-62]. Hence, it follows that hypoxemia in patients who have the overlap syndrome is more severe than that seen in either individual syndrome. These patients typically have baseline oxygen saturation values lower than normal subjects, and the greatest predictor of nocturnal hypoxemia is daytime hypoxemia [14]. Sanders et al. [24] examined the degree to which COPD and OSA independently and jointly contribute to oxygen desaturation during sleep. After adjusting for confounding factors, the OR for nocturnal oxygen desaturation was considerably increased in OSA. Bednarek et al. [63] compared polysomnographic variables between overlap syndrome and OSA patients. The overlap syndrome group had lower mean oxygen saturation and spent more time in oxygen desaturation than the OSA group.

A certain degree of hypercapnia occurs in normal subjects during sleep. Minute ventilation and CO_2_ sensitivity progressively decrease as the depth of sleep increases. It was shown that the slope of the hypercapnic ventilatory response decreases during NREM sleep and is more blunted in REM sleep [64,65]. This finding results from a change in the brainstem responsiveness to hypercapnia in NREM and REM sleep. COPD patients, having a mechanical disadvantage to increase tidal volume due to flattened diaphragms, demonstrate an altered hypercapnic ventilatory response during sleep. Patients who have coexistent OSA have repetitive acute hypercapnic events resulting from apneas and hypopneas, leading to arousal with stimulation to increase ventilation. When ventilation is not much compromised, CO_2_ drive will be preserved, and patients will remain normocapnic, while hypercapnia will develop when blunting takes place [51,66,67]. Moreover, hypoxia may also affect HCVR, since it interferes with synthesis and turnover of a wide range of neurotransmitters and shifts the slope of HCVR to the left. In addition, hypoxia may lead to a depression of hypoxic ventilatory responsiveness, leading to reduced ventilation and reduced CO_2_ elimination, both between apneas and in daytime [68]. Gold et al. suggested a reduction of HCVR and HVR due to chronic hypoxemia, worsening apnea, without recovery during wakefulness when oxygen was administered [69]. Leech et al. associated an improvement in oxygenation with a less depressed responsiveness of control centers, ameliorating the severity of OSA [70]. Moreover, it was reported that hypercapnia could develop in OSA in the absence of COPD [23], but also in overlap syndrome independently of lung function [71]. The observation that OSA patients with reduced respiratory drive do not increase their ventilation when terminating apnea could explain this development [72]. Effectively, daytime hypercapnia is more common in overlap syndrome as compared to simple OSA [23]. However, a correlation between hypercapnia and the frequency and duration of respiratory events during the night could not be observed [72,73]. A progressive resetting to a lower sensitivity threshold that occurs in the central chemoreceptors could be involved. Chronic stimulation bombarding the respiratory neuronal network could reset the chemoreceptor threshold, that appears over several years. The finding of an increase in slope as well as a left shift of the HCVR after tracheostomy could support this [74]. Finally, also constitutional or genetic factors may be responsible for lowered HCVR in hypercapnic patients [75]. Altogether, although recent and in-depth studies in overlap syndrome are lacking, available evidence supports more severe oxygen desaturations, worsened sleep quality and CO_2_ desensitisation as consequences of overlap syndrome. However, obesity as a confounding factor, giving rise to CO_2_ alterations, could not be excluded. This indicates that both syndromes cannot be completely separated, but are classified artificially based on strict and rigid definitions.

#### Pulmonary hypertension

OSA and COPD have been associated with variable degrees of pulmonary hypertension, defined as a mean pulmonary arterial pressure of ≥20 mmHg, potentially linked to the severity of disease. In one study [76], pulmonary hypertension was found in 13.6% of OSAS patients and in 80% of overlap patients. In another study the prevalence of pulmonary hypertension was 36% in patients with overlap, much higher than in usual OSA patients (9%), but somewhat lower than in the OHS [54]. The mechanism of pulmonary pressure elevation seems to be the relatively severe hypoxemia and not the inflammatory aspect of COPD or OSA. In general, subjects with COPD do not develop pulmonary hypertension, unless they have severe airway obstruction with substantial hypoxemia [77]. However, a small group of COPD patients will develop severe or so called “out-of-proportion” pulmonary hypertension, defined as a mean pulmonary artery pressure above 40 mmHg [77]. In OSA, AHI was a weak predictor of pulmonary arterial hypertension, whereas nocturnal oxygen desaturation was a more significant determinant of the presence of pulmonary hypertension. Subjects with the overlap syndrome can develop pulmonary hypertension with only mild to moderate obstruction [3]. This may result from the combined effects of both diseases contributing to hypoxemia and effects on pulmonary hemodynamics, with less of a contribution from the underlying mechanism from COPD or OSA [42]. Mechanisms underlying these interactions on the pulmonary vascular bed have further to be unraveled. Anyway, pulmonary hypertension is highly prevalent in overlap syndrome and seems more related to the hypoxemia level than to the AHI.

#### Cardiovascular

COPD and OSA are independent risk factors for cardiovascular events and their coexistence in overlap syndrome probably increases the risk. Shiina et al. reported an increase in arterial stiffness and significantly higher plasma BNP levels in subjects with overlap syndrome than in those with OSA alone [78]. Overlap was also associated with a markedly higher right ventricular mass index and right ventricular remodelling index compared to a COPD-only group [79]. Finally, among elderly patients, the presence of overlap syndrome is associated with a marked increase in risk of new onset atrial fibrillation as compared to the presence of OSA or COPD alone [80]. The mechanisms underlying cardiovascular risk are still unclear, but may involve systemic inflammation, endothelial dysfunction, and tonic elevation of sympathetic neural activity [2]. It is unclear whether the overlap syndrome carries additive or synergistic consequences of the inflammatory consequences of these two disorders. There is evidence of increased circulating CRP and IL-6 levels in COPD whereas in OSA obesity is a major confounding variable and the evidence of an independent relationship between OSA and CRP/IL-6 levels is less clear. Data also indicate an activation of TNF-α in COPD and OSA with hypoxemia being the key factor. Oxidative stress occurs in COPD and OSA and is associated with an increased production of reactive oxygen species, principally from leukocytes. However, confounding factors such as cigarette smoking and obesity also promote oxidative stress. Finally, there is evidence of activation/dysfunction of circulating leukocytes in OSA and COPD, which has particular relevance because leukocyte accumulation and adhesion to the endothelium are of key importance to atherosclerotic plaque formation [30]. Taken together, overlap syndrome has a substantial cardiovascular burden, in line with the burden described in isolated OSA or COPD. Whether coexistence in overlap syndrome is effectively associated with more inflammation, oxidative stress and activated cell lines compared to isolated OSA or COPD is an unanswered question. Again, an impact of obesity (hypoventilation) cannot be ruled out.

## Obesity hypoventilation syndrome

At the end of the spectrum of sleep-disordered breathing, which starts with simple snoring and evolves into OSA (eventually in association with COPD), OHS can be found. OHS is commonly defined as a combination of obesity (BMI ≥ 30 kg/m^c^) and awake arterial hypercapnia (PaCO_2_ > 45 mmHg) in the absence of other known causes of hypoventilation [4]. Patients are characterized by a spectrum of findings: episodes of obstruction, hypoventilation or sustained obstructive hypoventilation due to partial upper airway obstruction [5]. We don’t exactly know why some obese patients develop OHS, while others do not, nor do we fully understand the etiology of OHS, although it is almost certainly multifactorial in nature [6]. Patients may complain of fatigue or daytime sleepiness, but many remain asymptomatic with no sleep-related complaints. Sleep hypoventilation alone does not define OHS unless daytime hypercapnia is also present. It was hypothesized that obese patients with hypoventilation during sleep without awake hypercapnia have a “prodromal” form of OHS and will later develop chronic hypercapnia [81,82]. Some authors suggest that OHS is a mixed disorder of “can’t breathe”(unable to overcome impediments to breathing resulting from derangements in lung function, the performance of the respiratory muscles, and/or the mass loading effect of truncal obesity) and “won’t breathe” (decreased ventilatory drive disorder) [83-85]. Abnormal load responsiveness, ventilatory muscle dysfunction, increased respiratory work and CO_2_ production, impaired central respiratory drive and repeated airway obstruction during sleep are all possible pathophysiological components in this entity, but the precise contribution of each remains to be fully elucidated [5,6,86,87]. Different responses to CPAP, BPAP or NIV might reflect an intriguing possibility: the weight of the different pathophysiological mechanisms may vary in individuals with OHS. It seems that in some patients, severe OSA might be a major contributor to OHS pathophysiology, with respiratory system mechanics playing only a minor role. These patients could be successfully treated with long-term CPAP. On the other hand, other patients might stand out as having moderate or severe restrictive pulmonary defects and considerable nocturnal oxygen desaturation with low AHI values. These patients would require long-term NIV or BPAP ST/T. Controlled pressure support/control is necessary if CPAP fails [6,88-92].

The incidence of OHS increases significantly as obesity increases, with a reported prevalence of around 10 to 20% in outpatients presenting to sleep clinics [54,93-95] to almost 50% of hospitalized patients with a BMI greater than 50 kg/m^2^[96]. Current estimates suggest that around 0.15 to 0.4% of the population may have OHS [97,98].

### Development of obesity hypoventilation

**Figure 3:**
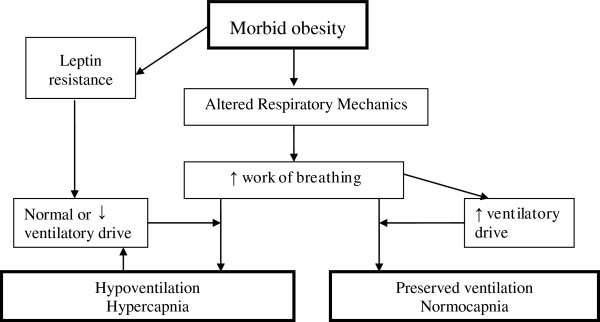
Interactions between the cardinal components of obesity and hypoventilation.

There are clearly specific differences between obese individuals which determine that only some morbidly obese subjects develop awake hypoventilation. The mechanisms underlying the selective development of awake hypoventilation is a major subject of interest in respiratory sleep medicine. Various compensatory mechanisms are adopted by morbidly obese subjects to maintain eucapnia, despite chronically loaded breathing [82], but are impaired or overwhelmed in OHS. The interactions between the cardinal components of obesity and hypoventilation are shown in Figure 3.

#### Ventilatory system mechanics

Obesity, particularly when it is severe, can be associated with significant changes in pulmonary mechanics and respiratory muscle performance. In simple obese, there are also data which show that the mechanical influences are of relatively low importance, with an approximate 0.5% decrease in VC, TLC, and RV with each unit increase in BMI, and an approximately 1% decrease in FRC and ERV for each unit increase in BMI [99]. However, many of these effects are magnified in those obese patients who develop OHS compared with equally obese individuals either without or with sleep –disordered breathing [27,100] (see Table 1). Obesity, with a higher degree of central fat distribution, acts as a mass load on the respiratory system [73], which implies both a weight placed on the respiratory apparatus as well as an increase in respiratory inertance [101-103]. Evidence exists that a central pattern of fat distribution is predictive of the impairments in pulmonary function more than BMI [103]. This leads to a significant reduction in total lung capacity, vital capacity, functional residual capacity, and increases in residual volume [104]. Breathing at abnormally low lung volumes changes the elastic recoil balance between the chest wall and lung [105,106], and is associated with increased lung resistance [107] and inspiratory muscle strength [108,109]. Respiratory system compliance has been shown to be around 20% less in eucapnic obese subjects compared to individuals who are of normal weight, and almost 60% less in patients with OHS [110]. Breathing at low expiratory volumes signifies breathing near the closing volume, and closure of the most dependent airways and air trapping will occur, resulting in expiratory flow limitation, microatelectasis and the development of intrinsic positive end-expiratory pressure (PEEPi) [100,111]. The fat deposit in the chest wall can modify the respiratory mechanisms and affects gas exchange, worsening ventilation-perfusion matching particularly in the supine position. Breathing at low volumes not only results in breathing in a less compliant portion of the pressure-volume curve with increased effort to overcome the respiratory system elasticity, but also in tidal flow limitation, small tidal volumes and higher respiratory rate compared to non-obese [112-114]. This strategy is thought to optimize the oxygen cost of breathing, but also increases dead space. PEEPi imposes an additional threshold load on inspiratory muscles before any inspiratory flow is generated. All together, this can lead to a threefold increase in the work of breathing, both in the sitting and supine positions [110,115]. OHS patients must maintain an increased oxygen cost of breathing (15% compared to 3% in nonobese subjects) as well, which may result in a relative state of respiratory muscle fatigue [110,116,117]. Maximal voluntary ventilation, a measure of ventilatory endurance, is reduced in simple obesity and further reduced in OHS [4,110]. Patients with OHS also have a higher upper airway resistance both in the sitting and supine position, when compared to patients with moderate-to-severe OSA with similar degrees of obesity and control subjects [51,118]. Similarities and differences in lung function characteristics between morbid obesity without or with hypoventilation are summarized in Table 1.

**Table 1:** Similarities and differences in lung function characteristics between morbid obesity without or with hypoventilation

	**Normocapnic morbid obesity**	**Obesity hypoventilation syndrome**
**Lung volumes:**		
**FRC**	↓	↓
**ERV**	↓	↓↓
**TLC**	Normal	Normal or ↓
**Respiratory compliance**	↓	↓↓
**Respiratory impedance**	↑↑	↑↑↑
**PaCO**_**2 **_**(awake)**	Normal	↑ or ↑↑
**PaO**_**2 **_**(awake)**	Normal or ↓	↓ or ↓↓
**HCO**_**3**_**-**	Normal	↑ or ↑↑
**Respiratory drive**	↑↑	Normal
**HVR**	Normal to ↑	↓↓
**HCVR**	Normal to ↑	↓↓
**P 0.1**	↑	↓↓
**MVV**	↓	↓↓
**Pimax**	Normal or ↓	↓ or ↓↓
**Work of breathing**	Sitting – normal to ↑	Sitting - ↑↑
	Supine - ↑↑	Supine - ↑↑↑
	Sleep - sitting - ↑↑	
	Supine - ↑↑	
**Upper airway resistance**	Sitting - normal	Sitting - ↑
	Supine - ↑	Supine - ↑↑↑

FRC: functional residual capacity, ERV: expiratory reserve volume; TLC: total lung capacity; HVR: hypoxic ventilatory response; HCVR: hypercapnic ventilatory response; MVV: maximal minute ventilation. ↑: mild increase; ↑↑: moderate increase; ↑↑↑: strong increase; ↓: mild decrease; ↓↓: moderate decrease.

The role of diaphragmatic weakness in the pathogenesis of this disorder remains uncertain, because patients with OHS can generate similar transdiaphragmatic pressures at any level of diaphragmatic activation compared to eucapnic obese subjects [116,117]. Pankow et al. have shown that non-invasive positive-pressure ventilation unloads the inspiratory muscles in patients with OHS [113]. These results emphasize the role of respiratory muscle fatigue in OHS [118,119]. Values of maximum inspiratory and expiratory pressures less than 70% predicted should prompt the clinician to consider OHS rather than simple OSA [100].

It could be speculated that respiratory muscle performance may be affected by the biochemical disturbance associated with hypoventilation (acidosis,hypoxemia, hypercapnia, inflammation). Hypercapnia is known to have deleterious effects on diaphragmatic function. Recently, Monneret et al. reported that insulin growth factor I (IGF-I) levels were inversely related to the vital capacity in OHS, an indirect marker of diaphragmatic strength. This indicates that IGF-I plays a role in the mechanical capacity of the diaphragm [120,121]. Moreover, the relationship between increased triglycerides and low IGF-I may represent one of the mechanisms involved in the OHS increased cardio-vascular risk, given that IGF-I is a well-established vascular protective factor. Another possible mechanism may be related to the metabolic syndrome and the low-grade inflammation associated with excess visceral and intrathoracic adiposity. Lin et al. have shown that metabolic syndrome was associated with a higher risk of restrictive lung impairment after adjustment for confounders (age, gender, BMI, physical activity, alcohol consumption) [119].

Thus, it is difficult to determine whether respiratory muscle fatigue is a cause or effect of OHS [122]. Whether a primary myopathic process also exists is currently unknown, as detailed muscle structural analysis has not been performed in subjects with OHS. In addition, the higher degree of central fat deposition compared with eucapnic obese patients results in a more pronounced cephalic displacement of the diaphragm. Compression of dependent zones and closure of small airways,which worsens in the supine position [123], and reduction of lung volumes and altering gas exchange will result to a much greater degree than weight deposited peripherally [103,124]. Improvement in VC and TLC can be achieved with positive pressure treatment, even without any significant change in weight [125]. Such improvements are explained by improved pulmonary compliance, secondary to a decrease in the closure of dependent airways and the opening of microatelectasis.

Therefore, it does not appear that obesity is the only determinant of hypoventilation, as only a minority of morbidly obese patients develop chronic hypercapnia [94,113]. A higher degree of central fat deposition, acting as a mass load, increased respiratory inertance, changes in the elastic recoil balance, lung resistance, inspiratory muscle strength, as well as air trapping, microatelectasis, V/Q mismatch, tidal flow limitation and increased work of breathing will finally result in a relative state of respiratory muscle fatigue. Which of these mechanisms will have most impact will differ in an individual patient.

#### Control of breathing and CO_2_ desensitisation

Obese subjects have increased rates of oxygen consumption (VO_2_) and carbon dioxide production (VCO_2_), even when they are at rest. Thus, obese persons must increase their minute ventilation to meet the increased oxygen requirements and maintain adequate alveolar ventilation [126,127]. The central drive to breathe is significantly higher in eucapnic subjects with morbid obesity than in normal-weight individuals. OHS patients fail to augment drive to compensate for the added load created by excess weight, permitting a gradual rise in CO_2_ to take place [73,116,118]. Central responsiveness to hypercapnia and hypoxia is blunted in OHS patients compared to normal weight subjects and eucapnic obese patients with or without OSA [52,95]. Patients with OHS can achieve eucapnia during voluntarily hyperventilation, implying that impairments in respiratory system mechanics alone do not explain the hypoventilation [86]. In simple obesity, the mouth occlusion pressure (P0.1 response) is also higher than seen in nonobese patients [116], but it is unclear whether this is applicable in OHS as well. The slope of the hypercapnic ventilatory response is < 1 l/min/mmHg in OHS, between 1.5 and 2.5 l/min/mmHg in eucapnic obese individuals and 2-3 l/min/mmHg in healthy subjects [51,128,129]. The decrease in ventilatory response is attributed to an inadequate increase in tidal volume as a result of a blunt neural response to hypercapnia [73,95], and has been demonstrated to improve with CPAP or BPAP [130,131] or not [132]. The hypoxic ventilatory response is also blunted in subjects with OHS. This abnormality is however not familial and improves with treatment as well [132,133]. Alterations in ventilatory responsiveness are also not as homogenous as initially thought [132] and are related to the daytime vigilance [134]. Individuals with low HCVR exhibit more daytime sleepiness, which is directly related to a higher percentage of REM sleep spent in hypoventilation [134]. A lack of relationship between BMI, HCVR and plasma bicarbonate was reported by Raurich et al., and with PaCO_2_ by Kessler et al., which might indicate on the other hand that obesity acts as a trigger toward hypoventilation in those patients who already have physiological abnormalities [54,135]. Gas exchange abnormalities will initially be confined to REM sleep, but over time buffering of the raised carbon dioxide produces a secondary depression of respiratory drive that will further reduce ventilation not only during sleep but during wakefulness as well [82].

An intriguing component of OHS pathogenesis concerns the metabolic consequences of obesity and its effect on ventilatory control. Leptin is a protein produced specifically by the adipose tissue and acts on the central respiratory centers to stimulate ventilation, whereas leptin deficiency has been associated with hypoventilation [136-139]. It has been hypothesized that elevated leptin levels may be a compensatory mechanism by which obese subjects remain normocapnic, but resistance to leptin may develop [140].

A possible mechanism could be central leptin resistance or reduced cerebrospinal fluid penetration of leptin. For leptin to affect the respiratory centre and increase minute ventilation, it has to penetrate the blood-brain barrier [141-143]. Also alteration at the level of the central receptor could be hypothesized [144,145]. In some obese subjects, central leptin resistance may lead to depressed ventilatory drive and, hence, OHS [146]. Campo et al. reported that higher serum leptin concentrations are also associated with both a reduced respiratory drive and a reduced response to hypercapnia [147]. Strikingly, leptin levels are a better predictor of hypercapnia than the degree of adiposity [148]. Yee et al. demonstrated that regular non-invasive ventilation use reduces leptin in OHS [87], although evidence is controversial [148].

Treatment of OHS with CPAP usually occurs concomitantly with a leftward shift of hypercapnic or, possibly, with an increase in the gain of both hypercapnic and hypoxic responsiveness, although a relationship between improvement in CO_2_ responsiveness and in PaCO_2_ may not always be recognized [130,131,149]. Weight loss by bariatric surgery improves hypercapnia and hypoxia [150], and HCVR [151]. This resetting in chemical drive could indirectly hint to a contribution of OSA to the onset of OHS.

Last but not least, the sustained hypoxia characteristic of sleep breathing in OHS may further contribute to the perpetuation and progression of abnormal breathing through impairment of the arousal response, further adding to the inability of these subjects to unload CO_2_ following apneic events [152-154].

The above mentioned changes in the respiratory control system of OHS patients make them more vulnerable to acute deterioration of their ventilatory systems when faced with new insults, such as chest infection or mild worsening of cardiac function. This makes OHS patients more susceptible to acute ventilatory failure [126,154]. Altogether, the interplay of respiratory control mechanisms and metabolic leptin alterations seems to be of utmost importance. These lead to inability to increase central respiratory drive, consequent CO_2_ accumulation, blunting of chemoreceptors and finally CO_2_ retention during the daytime. The precise mechanism on how leptin exerts its central effects is still questioned, but could be linked with leptin receptor gene variations and altered signal transduction [155-157].

#### Obstructive sleep apnea and upper airway patency

There is abundant evidence that OHS is tightly linked with sleep-disordered breathing, most commonly OSA [99]. Approximately 90% of OHS patients have underlying OSA [4].

Excessive fat deposition induces an enlargement of soft tissues surrounding the upper airway, compromising the pharyngeal airspace, and therefore predisposing the airway to closure during sleep [126]. Moreover, the reduced lung volumes reduced the inspiratory-related caudal tracheal traction that stabilizes upper airway structures [158]. Also fluid shifts from the legs to the neck during sleep contribute to the pathogenesis of OSA [44]. OSA could predispose to daytime hypercapnia by causing nocturnal hypoxemia [68] and sleep fragmentation [159] that, in turn, impair mass load compensation thereby predisposing obese patients to blunting of the respiratory drive and hypercapnia [4,72]. This was supported by studies that have correlated nocturnal desaturation (but not AHI) with hypercapnia [160]. Loss of the so-called normal CO_2_ response to apnea that protects against the development of hypercapnia by stimulating respiratory compensation for each apnea during the interapnea period is thought to predispose to daytime hypercapnia in patients with OSA [59,161]. Patients with these concurrent syndromes may be caught in a “vicious circle”, which may lead to more severe exposure to hypoxemia and hypercapnia and further attenuation of the ventilatory response [4]. However, there are some conflicting data regarding the impact of OSA. In a series of 219 patients with OHS, the only variables that correlated with a decreased HCVR were high daytime PaCO_2_ and older age in men, whereas in women an elevated BMI correlated with an increase in HCVR [162]. A relationship between HCVR and OSA could not be demonstrated, while another study showed that the severity of OSA based on AHI correlated with hypercapnia [163].

The remaining 10% of patients with OHS have an apnea-hypopnea index less than 5 [164,165]. The sleep-disordered breathing in this subset of patients has been labelled as sleep hypoventilation and is defined as an increase in PaCO_2_ during sleep by 10 mmHg above wakefulness or significant oxygen desaturation that is not explained by obstructive apneas or hypopneas [164]. It was shown that a significant proportion of these patients diagnosed initially with isolated sleep hypoventilation will later exhibit OSA if withdrawn from non-invasive ventilation [166]. The development of awake hypercapnia has been shown to correlate strongly with the proportion of sleep time spent less than 90% SaO_2_[97]. However, it is probably not the hypoxemia, but the underlying hypoventilation that leads or aggravates hypercapnia with a spill over during the daytime when desensitisation occurs. On the other hand, again, hypoxia could interfere with the synthesis of a number of neurotransmitters involved in central respiratory control, including γ-aminobutyric acid, dopamine and adenosine, and hence with worsening of sleep hypoventilation [167]. Summarized, upper airway patency is often impaired in obesity due to excessive fat deposition, reduced caudal tracheal traction and fluid shifts, promoting the development of OSA. OSA in itself can be associated with hypercapnia by causing hypoxemia, sleep fragmentation and depressed CO_2_ drive.

#### Model combining sleep-disordered breathing, central respiratory drive and renal buffering

The role of OSA in the pathogenesis of hypoventilation has been established by the resolution of hypercapnia in the majority of patients with OHS treated with CPAP or BPAP without any significant change in BMI. There is pathophysiologic basis behind how severe OSA, as measured by AHI, could lead to hypercapnia [66,168,169]. In patients with OSA, the minute ventilation during sleep does not decrease due to the large increase in the minute ventilation between the obstructive respiratory events. Obstructive apneas can, however, lead to acute hypercapnia if the duration of the inter-event hyperventilation is inadequate to eliminate the accumulated CO_2_[169]. In cases of very severe OSA, there is not much time left to increase the minute ventilation between the obstructive events and to off-load CO_2_, leading to significant nocturnal hypercapnia. This acute hypercapnia causes a small increase in serum bicarbonate level that is not corrected before the next sleep period if the time constant of bicarbonate excretion is longer than that of CO_2_. Because the bicarbonate in the serum (and brain tissue) rises during the chronic, compensated respiratory acidosis, the baseline pH of the cerebrospinal fluid is actually well defended, so the pH and the stimulus for the brainstem chemoreceptors are not much changed in the steady state. However, should the CO_2_ level rise further, elevated bicarbonate level will blunt the ventilatory response to CO_2_ by reducing the change in pH associated with the change in CO_2_, and the chemosensory stimulus will be less for any given change in the level of CO_2_[170]. This would ultimately result in a higher wake CO_2_ level [113,168]. In a recent study it was confirmed that patients with higher bicarbonate concentration had a more blunted CO_2_ response [135]. The development of decreased central respiratory drive and/or the greater ventilatory limitations imposed by more extreme central obesity may initiate the acute failure to compensate for OSA, but only if the renal compensatory mechanism is impaired [100,171]. In other words, whether or not daytime chronic hypercapnia develops depends on the individual’s ability to unload both CO_2_ and bicarbonate during the waking period [168]. Thus, deficient renal bicarbonate excretion seems to play a critical pathophysiologic role, leading to a cumulative effect over subsequent periods of sleep, which eventually results in a self-perpetuating state of chronic hypercapnia. Hence, the presence of obstructive apneas can trigger a vicious circle of acute hypercapnia, blunting the ventilatory response to CO_2_, but only if the renal buffering capacity is impaired. Concommittant presence of morbid obesity and airway obstruction could also lead more easily to limitation of the interapneic ventilation. This could represent a common mechanism by which COPD and obesity could predispose to the development of hypercapnia in OSA.

#### Neurohormonal changes

Besides leptin, adipose tissue is able to express numerous other adipokines, that are involved in energy homeostasis, as well as in vascular and endothelial physiology. These adipokines are thought to be the mediators of endothelial injury and atherosclerosis [172]. Although most adipokines promote insulin resistance and endothelial dysfunction, adiponectin (with insulin sensitizing and antiatherogenic effects) protects against these disorders. Adiponectin levels are decreased in obesity [173] and inversely correlated with cardiovascular morbidity. With both systemic hypoxia and tissue ischemia, adipokine levels are altered, with downregulation of adipokine expression and upregulation of leptin through HIF-1α activation [174,175]. Recently, Borel et al. reported that OHS is associated with a specific increase in the pro-atherosclerotic RANTES chemokine, a decrease in the anti-inflammatory adipokine adiponectin and impaired endothelial function. These three conditions are known to be strongly associated with an increased cardiovascular risk [176]. Based on these findings, OHS seems a specific cluster in obesity associated with specific inflammation and aggravated endothelial dysfunction. Moreover, they proposed to distinguish subclasses of inflammation among obese populations reflecting different risks and allowing tailoring specific anti-inflammatory treatments. Hence, the role of nocturnal hypoxia resulting from nocturnal hypoventilation and recurrent apneas, when present, may be critical. It might explain, at least partly, the excess of morbidity and mortality occurring in OHS [176]. Moreover, non invasive ventilation, the current first line therapy of OHS, should now be evaluated, in randomized controlled trials, not ony regarding its effects on PaCO_2_, sleep and quality of life, but also for its cardiovascular and metabolic impact [176]. These data illustrate the complex picture of OHS, and its cardiovascular consequences. However, adipokines other than leptin do not seem to be involved in the pathogenesis of OHS.

## Differences and similarities between overlap syndrome and obesity hypoventilation

Although overlap syndrome and OHS are very different conditions, they share some pathogenetic and clinical aspects [177], and represent substantial morbidity and mortality (Table 2). On the one hand, from the pathophysiologic point of view, the presence of COPD does not favour the occurrence of OSA and vice versa, and hence the occurrence of overlap syndrome is by coincidence [24]. In OHS, on the other hand, there is abundant evidence that it is tightly linked with OSA [4,100], and only a minority has nonobstructive sleep-disordered breathing [66,100]. However, overlap patients have important sleep-related oxygen desaturation and represent a high risk of developing hypercapnia and pulmonary hypertension, even in the presence of mild to moderate bronchial obstruction. OHS patients will, by definition, present always with respiratory insufficiency, and are therefore more often at risk for morbidity and mortality than the overlap population. Both disorders share complex interactions, among which are increased work of breathing related to (central) obesity, alterations of ventilatory drive, various associated sleep breathing disorders and neurohormonal changes such as leptin resistance. However, those with overlap syndrome do not necessarily present with obesity, and the ventilatory drive can be normal, enhanced or reduced [50,51] and the presence of hypoventilation is optional.

**Table 2:** Similarities and differences between overlap syndrome and obesity hypoventilation

	**Overlap**	**Obesity hypoventilation syndrome**
**Coexistent OSA**	No causal relationship	Causal relationship in 90% of the cases
**Hypoventilation (PaCO**_**2**_ **≥ 45 mmHg)**	Occasionally	Always (by definition)
**Obesity (BMI ≥ 30 kg/m**^**2**^**)**	Often (but not obligatory)	Always (by definition)
**Coexistent COPD**	Always (by definition)	Never (exclusion by definition)
**Coexistent restrictive pulmonary disease**	Occasionally mixed pattern	Often present
**FRC**	Decreased	Decreased
**Chemosensitivity (HCVR)**	Normal, enhanced or decreased	Decreased
**Leptine resistance**	Present	Present
**Hypoxemia pattern**	Intermittent (intermittent and chronic in severe cases)	Intermittent and chronic (90%), or chronic (10%)
**Level of hypoxemia**	Absent to very severe disturbance	Moderate to very severe disturbance
**Prevalence in general population**	1-4%	0.37%
**Prevalence in OSA**	10%	14%
**Pulmonary hypertension**	+ to ++	++ to +++
**Health care consumption**	Increased	Increased
**Mortality**	80% in 12 years	23% in 1.5 years
	90% in 8 years in LTOT	

+: mild increase; ++ moderate increase; +++severe increase.

The interindividual differences are intricate, reflecting phenotypic complexity and admixture with various other respiratory diatheses (e.g. smoking). In OHS, pathogenetic factors like obesity related decrease in chest wall compliance do not in general appear to be sufficient to cause OHS as they are present in many non-hypercapnic obese individuals. However, when minute ventilation falls below a range necessary to compensate for the metabolic demands, hypercapnia will result.

These mechanisms can play a role in some obese patients with overlap syndrome as well, but hypoventilation will finally emerge when compensatory mechanisms of CO_2_ homeostasis fail or become overwhelmed [67,178,179]. Both syndromes share a high prevalence, namely 10 to 20% for OHS in patients with OSA [4,96,100], while COPD is reported in about 10% of OSA patients, with some studies even reporting higher figures [20-24]. The prevalence of overlap and OHS in the general population is estimated to be 1% and 0.37% respectively [20-23,96-98]. Both overlap and OHS patients can present with the typical symptoms of OSA, but depending on the complexity of the syndromes, some particularities can be observed. Those with OHS can have characteristic symptoms, on the one hand, due to elevated PaCO_2_, and consist of cognitive impairment, daytime hypersomnolence and morning headache. Chronic hypoxemia during wakefulness, on the other hand, leads to signs of cor pulmonale, pulmonary hypertension, polycythemia, and respiratory failure. Sleep-disordered breathing is most often present with symptoms of loud snoring, fragmented sleep, gasping, nocturia, fatigue and non-restorative sleep. The clinical manifestations of OHS depend on the degree of obesity, the presence of comorbidity like OSA and, of course, on the degree of hypoventilation. Overlap patients can also present these symptoms, depending on the relative contribution of the two components to the syndrome, and the eventual development of respiratory failure. In OSA patients with advanced stage COPD, dyspnea, orthopnea and peripheral edema are also common and can be the dominant symptoms, while only a minority of overlap patients will develop respiratory insufficiency. Therefore, it can be estimated that these symptoms will be less common than in OHS. In both disorders, also a high prevalence of pulmonary hypertension was reported. Closely related to the most often more severe hypercapnia and hypoxemia during the daytime found in OHS, these patients present with a higher prevalence of pulmonary hypertension [88,109,135,180]. However, thresholds for hypercapnia/hypoxemia severity and duration necessary to give rise to sleep-related symptoms or cardiovascular complications, such as pulmonary hypertension, in individual OHS patients, are unknown. The change in evening to morning PaCO_2_ has been shown to be highly correlated with severity of sleep hypoventilation, and could be used as a marker [181]. In patients with overlap syndrome, also no cut-off values have been defined that warrant treatment, but an AHI ≥ 15 has been shown in isolated OSA to be clinically relevant [182]. This criterion could be applied in both syndromes [183]. Overlap syndrome as well as OHS also share a higher morbidity [7,17,54,76,78-80,135,170,181],[182] and mortality [16,26,96,184,185], decreased quality of life [58,186], and an increased use of health care resources [187,188]. OHS is associated with chronic heart failure, angina, arterial hypertension, cor pulmonale and endothelial dysfunction, while overlap syndrome is associated with arterial hypertension, new onset atrial fibrillation and cardiovascular remodelling. Patients with OHS tend to use more antihypertensive drugs, have higher insulin resistance, and are more likely to be treated with antidiabetic agents [16,135,164,187]. Overlap patients also more often present with COPD exacerbations than simple COPD patients (relative risk of 1.70) [16] and show a trend to less prednisolone use after treatment [28]. OHS patients who refused treatment with non-invasive ventilation, had a mortality rate of 46% over an average follow-up period of 50 months [88]. In overlap, reduced survival was reported in those refusing CPAP therapy (relative risk of 1.79) [15,184]. Chronic daytime hypercapnia would emerge if both the acute ventilatory compensation for transient nocturnal hypercapnia is compromised, as well as the bicarbonate excretion, as might be seen under condition of hypoxia (f.i. chest infection), diuretic therapy, or heart failure [170,178]. This makes both patient categories more susceptible to acute ventilatory failure [126]. Despite this, the diagnosis of overlap syndrome and of OHS appears to be often overlooked, especially in a clinical setting when dealing with the other illnesses of these patients [189]. The evidence however supports that recognition and treatment of both diseases is imperative.

## Conclusion

Overlap syndrome and OHS are not rare conditions, due to the high prevalence of COPD, obesity and OSA. They are both serious medical disorders associated with significant morbidity and mortality. Its diagnosis can have a significant impact on an individual subject and on the health care system as a whole. Both conditions present unique characteristics which set them apart from either COPD, obesity or OSA. They share some common pathways, and hypercapnia will develop when the normal compensatory mechanisms that should normally operate to maintain ventilation despite respiratory system abnormalities are impaired. Obesity is generally believed to be the major etiologic factor responsible for both syndromes in the majority of the patients. The interaction between the components of these two diseases is not completely disentangled, and may reflect phenotypic complexity and admixture with various other respiratory diatheses. Factors that compromise the acute ventilatory compensation for the transient sleep hypercapnia, altered bicarbonate excretion and respiratory mechanics finally determine the development or deterioration of hypoventilation. Recognition of sleep-related disturbances and a better understanding of their interactions in subjects with COPD and in subjects with obesity will allow to optimize management of these patients and their quality of life.

## Competing interest

The authors declare that they have no competing interests.

## Authors’ contributions

JV and WM conceived of the study, and participated in its design and coordination and drafted the manuscript. Both authors read and approved the final manuscript.
